# Consumer Understanding of Food Quality, Healthiness, and Environmental Impact: A Cross-National Perspective

**DOI:** 10.3390/ijerph17010169

**Published:** 2019-12-25

**Authors:** Dacinia Crina Petrescu, Iris Vermeir, Ruxandra Malina Petrescu-Mag

**Affiliations:** 1 Faculty of Business, Babes-Bolyai University, 7 Horea Street, 400174 Cluj-Napoca, Romania; 2 BE4Life, Department of Marketing, Innovation and Organization, Faculty of Economics and Business Administration, Ghent University, 9000 Ghent, Belgium; iris.vermeir@ugent.be; 3 Faculty of Environmental Science and Engineering, Babes-Bolyai University, 30 Fantanele Street, 400294 Cluj-Napoca, Romania; malina.petrescu@ubbcluj.ro

**Keywords:** food quality evaluation, food quality cues, food healthiness, food environmental impact, consumers, Belgium, Romania

## Abstract

The last few decades testify that consumers’ concerns for healthier lifestyles and environment care are driving forces for reshaping food buying intentions and their perspectives on food quality. The present study identifies the importance that consumers attach to quality, health, and environment selected cues of purchased food products. More precisely, to elicit preferences for social, environmental, and qualitative food cues, a survey instrument was developed and applied on 797 Belgian and Romanian consumers. Our findings suggest that investigated consumers most frequently use freshness, taste, and appearance to evaluate food quality. The use frequency of food quality cues related to health is primarily influenced by the attention paid to food quality. The most relevant cues of food healthiness are ingredients, nutrition facts, and additives and for food environmental impact are packaging, food origin, and production type. It is concluded that food quality receives high attention both from Belgian and Romanian consumers and health and environment related cues can be used as a means of improving consumer health and environmental protection.

## 1. Introduction

The demand for high quality food has constantly increased during recent decades, as has the interest in the food quality issue both in response to market pressure (such as requests from increasingly demanding and knowledgeable consumers) and also as a reaction to other factors, for example health and environmental concerns [1]. The consumer of the 21st Century is a highly demanding one, exhibiting greater concern about quality and health benefits with respect to products he/she buys [2]. Food quality is a central issue in today’s food economics [3], and the last few decades testify that consumers’ concerns for healthier lifestyles and environment care are driving forces for reshaping food buying intentions and their perspectives on food quality.

Based on the premise that food quality is formed by the perceptions held by consumers [4], the present contribution draws on the assumption that one important key to the understanding of the food quality evaluation is the discovery of the cues used by consumers in this process. This understanding of consumers’ perceptions of food quality is highly relevant because their buying decisions depend on these frames [5]. The situation is complicated even more when consumers’ interpretation of quality contradicts the official definition of it; that is, when perceptions create barriers in recognizing food quality [6] or when consumers’ interpretations generate a quality perception for food products that do not qualify to receive it. Consequently, based on these assumptions, it is self-understood that differences in food quality perceptions appear over time and places. This complex and dynamic character of food quality requires its constant investigation to capture as much as possible its current meaning.

Generally, quality evaluation of food consists of two stages. The first one, which is the focus of the current study, precedes the purchasing act, and the second one is related to what happens after the purchasing act while consuming the food. Regarding the first stage, at the point-of-purchase, consumers use both explicit cues (e.g., color, price, and claims) and subtle cues that are communicated by packaging design, like graphic design, material, and color [7]. Europeans value food labels as one of the most trusted sources of information [8], and this reality is further strengthened by research that reports that consumers’ perceptions of product quality are to a high degree affected by information [9,10]. Markets need reliable information in order to be functional. The existence of information asymmetries (when one side knows more about a product than the other) is usually attributed to the failure of government to create proper legislation and enforce its compliance, business actors noncompliance with existing regulations, lack of consumer attention to or understanding of the available information, and industry marketing practices [11]. Undoubtedly, labelling is one of the instruments that helps consumers make a well founded choice [12,13]. Many studies consider labelling as a means of aggregated communication of environmental and health product features [14,15,16]. A smaller informational gap between producers and consumers (e.g., through the provision of detailed product information, in this case on environment and health cues) and its associated perceived usefulness are largely reported to affect consumer behavior positively by increasing consumer trust through the provision of detailed, credible, and transparent signals [16,17,18].

Consumers are generally unable to assess the quality of food without the reflection of different traits [19], and as long as labels display ingredients, expiration date, health information, and environmental attributes, consumers often rely on them when they assess the food quality attributes [20]. This study scrutinizes our understanding of which cues signal food quality for Belgian and Romanian consumers. Moreover, concerns about food health are among the main motivators of consumers to purchase different types of products, but the literature on consumer preferences does not investigate what “health” means to these consumers [21]. To this end, we undertook a qualitative study to identify the importance that consumers attach to attributes of purchased food products that indicate health and environment impact. However, since health and environmental friendliness are invisible product characteristics, health and environment protection attributes must be deduced from more concrete intrinsic and extrinsic cues [22]. The main objectives of the study are to find out which are the main cues used by consumers to evaluate food quality, healthiness, and the environmental impact of food and to reveal what factors influence the use frequency of health related food quality cues. Therefore, of special interest for this study is to highlight the place of the cues related to health and environmental protection within the diverse landscape of cues used by consumers to define food quality, due to the high importance that concern for health and for the environment has gained during the last few decades [23,24]. Thus, special emphasis is placed on testing the relevance of health and environmental cues in consumers’ food quality evaluation. As long as there is an evident interest that targets the changes in consumers’ food expectations and preferences, the translation of consumers’ food quality perceptions into an instrument usable in marketing actions is critical for stakeholders preoccupied with what elicits consumers’ satisfaction in terms of food quality cues. It is self-understood that food quality perception is a complex process within which the use of quality cues represents only one important stage among others, but discussing them all is not the scope of this paper.

The main novelty of this study relies on revealing which are the food quality cues most frequently used by consumers. Furthermore, the study empirically establishes which are the cues used by consumers to evaluate the health and environment characteristics of food, thus informing sustainability related marketing theory and practice. A third element of originality stands in casting light on a set of variables that can predict the use frequency of several food quality cues related to health (ingredient list, fat content, salt content, and sugar content).

Comparatively, a similar study by Mascarello et al. [1] aimed to discover the cues that contributed most to defining the quality of a food product. The difference from the present research consists of the number of tested cues (their study tested nine quality elements) and the fact that it identified two consumer groups according to the quality cues that they used. Brecic et al. [25] analyzed the importance of quality cues to consumers, but the number of cues investigated in their study was lower compared to the number of cues tested in the present study (20 vs. 59). 

Other studies investigated the influence of one or more specific variables on food quality perception [9] and not the general perception of food quality as happens in the present research.

Regarding the focus on Belgian and Romanian consumers, this is the first study to scrutinize the use frequency of quality, health, and environmental cues by these consumers. The paper of Januszewska et al. [26] was the only one that reported the findings of a comparative analysis between these two countries (in addition to Hungary and the Philippines); however, that was done in relation to a different topic, namely consumers’ motives in food choice, using the Food Choice Questionnaire.

## 2. Conceptual Framework

As posited by Lancaster [27], Kramer and Twigg [28], or Molnar [29], food quality is the assemblage of attributes (such as physical properties, chemical composition, sensory attributes, microbiological and toxicological contaminants, shelf-life, packaging, and labeling) that determine the product’s performance, are in dynamic interrelation, and influence the consumer in accepting or rejecting the product. According to Zeithaml [30], the perceived quality is seen as “the consumer’s judgment about a product’s overall excellence or superiority”. Product characteristics used by consumers to define food quality are not only numerous, but also dynamic, changing according to consumers’ interests, concerns, needs, or knowledge. During recent years, consumers started to assign higher importance to the sustainability aspects in their food buying decisions [31]. For instance, since the concern for environmental protection and food safety aspects has increased, many quality food characteristics have been associated with what takes place on farms and in the distribution chain (“from farm to fork”) and how crops and livestock husbandry were run [32,33,34]. Therefore, food safety, packaging, process, and nutritional value are among the most investigated food product quality attributes [35,36,37], and the care for the environment and sustainability [38,39] are increasingly retained for analysis. This trend justifies the inclusion in the questionnaire of health and environmental aspects. 

The elements that consumers use when they evaluate food quality have received different names in the literature, and they have been classified into specific groups according to their nature or the context of their utilization. Thus, Nelson [40,41] named them “qualities”, and he made the distinction between the search and experience qualities of a good. Search qualities are those that the consumer can determine prior to the purchase of a good through direct examination, such as a tomato’s color (for unpacked tomatoes), and experience qualities are those that the consumer cannot determine prior to purchase, but only when the product is used, as is the case for ice cream taste. Darby and Karni [42] added credence qualities, defining them as those qualities that cannot be evaluated in normal use and require additional costly information, such as the lower negative environmental impact of organic apples. Later, Ford et al. [43] used the term “attributes” and explained that credence attributes depend on the average consumer’s level of expertise, i.e., his/her technical expertise to assess the real performance of the product. The search-experience-credence (SEC) theory has been largely used in consumer behavior research, both for food and non-food items A reference framework to explain consumers’ food quality perceptions is the cue utilization theory by Jerry Olson and Jacob Jacoby [44], where it is argued that consumers resort to intrinsic and extrinsic cues to infer the quality of a product. The intrinsic ones are those that cannot be changed without altering the physical characteristics of the product (e.g., color, shape), and the extrinsic ones are all the others (e.g., price, brand, selling place). Often, intrinsic quality cues (e.g., taste, color, freshness) were found to be much more relevant than the external ones (price, brand, packaging) in determining consumers’ overall quality perceptions of food products [45]. 

The term “cue” is used with different meanings in the literature. For instance, Caputo et al. [46] used the concepts “cue attribute” and “independent attribute”. The former is an intermediary element that conveys information about another attribute (e.g., a “natural” product tells something about the “healthiness” of that food), and the latter transmits information by itself (e.g., being fried or raw). However, there are situations when an attribute can be both a “cue” and “independent”, depending on personal interpretations and context. Steenkamp [47] made a distinction between quality cues and quality attributes, defining the former as what consumers observe and the latter as what consumers want. Thus, quality attributes can usually be verified only after purchasing and consumption, which determines that consumers use quality cues at the purchasing time. In the total food quality model, Grunert et al. [48] employed the term “cues”, and he identified two moments for their use, that is for quality evaluation (before purchase, when consumers form quality expectations, and after purchase, when consumers undergo the quality experience). Other studies, which investigated food quality evaluation by consumers, also used the term “cues” [49,50,51]. The current study investigated food quality evaluation at the purchasing moment; therefore, we adopted Steenkamp’s [48] definition, and the term quality “cues” was used.

Our review generated 59 items for quality evaluation. The synopsis of the cues with the corresponding studies where they were mentioned is included in Table 1.

Ajzen [103] demonstrated that consumers’ behavior is influenced by their perception regarding their ability to perform a specific behavior. Consequently, consumers’ perception of their capacity to correctly assess food quality was tested in the present study. Furthermore, besides the investigation of cues that indicate food healthiness, the use of food itself as a means to influence health was considered important in understanding the use of food quality cues related to health, and consumer perception of their capacity to influence their health through food was studied. In the present study, health oriented food quality was valued as how consumers perceived a food product would affect their health, and this orientation included functional qualities of foods. Health related qualities were mostly credence characteristics, because the impact of specific food consumption on one’s health is a matter of trust, and it can rarely be ascertained after consumption [22]. Not to be ignored is the fact that the concept of health can be understood from different scientific perspectives, including medical, nutritional, social, and psychological, and according to Brunsø et al. [46] for a consumer, health may imply two main dimensions: the first one concerns nutritional aspects (e.g., functional food, less fatty food, no GMOs), and the second is related to food safety and risk related issues, which was not a subject of investigation in this research.

## 3. Materials and Methods 

A wide variety of instruments has been used in practice to explain how consumers evaluated food quality, and they usually contained experience qualities such as taste and credence qualities like environmental impact and healthiness [104]. Following Power et al.’s [105] approach, the questionnaire used here contained items from a variety of studies that investigated attributes used by consumers to evaluate food quality and also several items included specifically for this study. Thus, the questionnaire was created based on the results of a literature review and of two focus groups, which generated together a list with 59 quality cues (Table 1). The inclusion of a large number of quality cues in the questionnaire was pursued to capture as much as possible consumers’ patterns of food quality evaluation. 

The conceptual framework for this study was adapted from the literature as follows. The authors developed a systematic search in electronic databases (e.g., Cambridge Journals, Emerald Management Journals 200, ProQuest, Science Direct Freedom Collection-Elsevier, Springer, Wiley Journals, and Google scholar). Papers containing the following terms and their combinations were searched: food, quality, consumer, evaluation, model, perception/perceived, cues, and attributes. Around 400 manuscripts were initially retrieved based on several criteria: English language, peer reviewed, and presence in journals with an impact factor. Out of these, half were selected for a deeper analysis. Firstly, the most cited food quality evaluation models were analyzed, and the quality cues present in them were extracted. These were: quality perception process conceptual model [47], perceived quality [106], Food Choice Questionnaire (FCQ) [107], food quality [108], and the total food quality model [46]. Secondly, a new analysis was made of the rest of the papers, which were published between the years 2010 and 2020, and additional cues were selected from these. Furthermore, exploratory research was carried out using a focus group in each country (Belgium and Romania) during which participants were asked to explain what cues they used to evaluate food quality. Finally, the cues list from the literature and the one from the focus groups were integrated into a final list (Table 1) based on which the authors developed the questionnaire. The questionnaire was pre-tested two times on groups of 30 consumers and revised before data collection. The questionnaire had two sections. 

In the section dedicated to food quality, firstly, the level of attention paid to food quality was assessed on a 7 point scale (1 = not at all, 2 =…, 3 =…, 4 = average attention, 5 =…, 6 =…, 7 = a lot of attention). Then, an open ended question was aimed at capturing consumers’ unaided awareness of the cues that are most frequently used by consumers for evaluating food quality. This question preceded the list with quality cues and requested that they indicate a maximum of five cues. Secondly, they received a list with the 59 quality cues, and they were requested to indicate how often each of them was used for the evaluation of food quality. A 7 point scale with answer options was attached to this question (1 = you took into account this information very rarely/never when you bought food, 2 =…, 3 =…., 4 = you took into account this information in about half of the cases when you bought food to evaluate its quality, 5 =…, 6 =…, 7 = you took into account this information very often/always when you bought food). Out of the 59 cues, at least 34 could be related to health and environmental aspects. 

Two open ended questions were asked to find out which cues were used by consumers to evaluate the healthiness of food and the environmental impact of food. A maximum of five cues was allowed to be mentioned in each case. 

Another question assessed consumers’ perception of their capacity to evaluate food quality reliably. For a deeper understanding of this perception, a comparative context was used in this question by asking consumers to evaluate also the reliability of food quality information obtained from another five additional information sources: “Producers of the food you buy, through their website”, “Producers of the food you buy, through the information on the label and package”, “Sellers/shopping assistants in the shops where you buy the food”, “Your family and friends”, and “Other consumers”. These five sources were selected through the focus groups. The answer options for the reliability of all these six information sources ranged on a scale from 1 to 7 (1 = no confidence at all, 2 =…, 3 =…., 4 = average confidence, 5 =…, 6 =…, 7 = complete confidence). Following the same reasoning, consumers’ perception of their capacity to influence their health through food consumption was studied by taking into account three possibilities: their power to maintain their current health state by eating the right foods, to improve their health state significantly by eating the right foods, and to damage their health significantly by eating improper food. The answer options were 1= no power to do so, 2 =…, 3 =…., 4 = average power, 5 =…, 6 =…, 7 = very high power to do so. 

The final section of the questionnaire collected socio-demographic data: gender, height and weight (used to calculate the body mass index (BMI)), health status (current or previous existence of serious health problems of consumers or of family members), and country of residence (Belgium or Romania). 

In total, 797 valid questionnaires were collected: 441 from Belgium and 356 from Romania. It was suggested that 5–10 responses per each estimated parameter would result in an appropriate sample size [109]. Therefore, the sample size used in this study was sufficiently large for analyzing the 59 item scale, providing a ratio of 13.5 cases per variable. According to Tabachnick and Fidell [110] who cited Comrey and Lee’s [111], a sample size of over 300 is good, as they gave the following indications: 50 cases is very poor, 100 is poor, 200 is fair, 300 is good, 500 is very good, and 1000 or more is excellent. The questionnaire was posted online on the isondaje.ro website and distributed in Belgium and Romania; also, a printed version was completed through face-to-face interviews by Romanian consumers. Everybody participated in the study voluntarily. The sample structure is presented in Table 2.

Several different statistical methods were used to understand how consumers evaluated food quality. These included descriptive statistics, bivariate analyses (Mann–Whitney U test and Wilcoxon signed rank test), and regression analysis. Standard linear regression was performed using SPSS to test the relationship between the attention paid to food quality, confidence in self-capacity to evaluate food quality, the power to maintain, improve, and damage health through food choices, and BMI (independent variables), on the one hand, and the use frequency of food quality cues related to health (dependent variable). The cues tested were: ingredient list, fat content, salt content, and sugar content. Analyses were performed with the software Excel and SPSS. 

## 4. Results and Discussion

Food quality received high attention from consumers, reaching 78.3% of the maximum level, with 5.7 points. The average score for the Romanian sample was 5.9 and for the Belgian one 5.5. A statistically significant difference was indicated by the Mann–Whitney U test between the Romanian and Belgian samples, with higher attention from Romanians. No statistically significant difference was generated by the existence of health problems of the respondent or of his/her family, and no correlation was observed between the attention paid to food quality and the BMI. Men and women did not statistically differ regarding the level of attention paid to food quality.

Relying on the assumption that not all 59 food quality cues were used in food quality evaluation at the same time, the answers to an open ended question about the most frequently used cues for food quality evaluation helped in discovering the most important ones. The number of quality cues mentioned by tested consumers in the open ended question was high (a total of 3183), covering 80% of the maximum number of answers (when all 797 respondents would have named five cues). They were aggregated into 39 groups, detailed in Table 3. 

The present research revealed that “appearance”, “price”, and “ingredients” formed the winning trio that dominated consumers’ judgment concerning food quality (Table 3). This finding was in agreement with previous studies that highlighted one or more of these cues as the most important in food quality evaluation [1,112]. Similarly, a study on Malaysian consumers highlighted that freshness and price were two of the most used quality cues for fresh meat, fruits, and vegetables, and freshness was judged by the physical appearance of the meat [50]. The price can be regarded as a motive to purchase, as an indicator of quality, and as an indicator of socio-economic status [113]. However, when specific products are investigated, other or additional cues stand out in consumers’ evaluation process. For example, when purchasing fresh meat, color, origin, and buying place seem to be the most frequently used search quality cues at the point-of-purchase [114,115]. When it was about locally grown food, freshness was among the first mentioned attributes [116]. For fresh meat, fruits and vegetables, freshness, price, and cleanliness were the main quality cues considered. Still, for the present investigation, appearance stood out compared to all the rest, being mentioned by more than half of the sample and having the highest difference from the next position compared to all other cues (20.3% above the next position, price, which gathered only 38.3% of nominations; Table 3). Within the 52.7% of consumers who relied on appearance in food quality evaluation, 43% of them mentioned the general term “appearance”, 8% indicated the “color”, and 1.7% of them the “shape”. Regarding the use of appearance within the two national groups, Romanians used it more often than Belgians (55.7% of the Romanian sample compared to 46.6% of the Belgian sample), and also, they mentioned color (15.9% vs. 2.6%) and shape (4% vs. 0.3%) more often than Belgians. It can be inferred that, among tested consumers, the appearance was more important in Romania compared to Belgium. 

Results on the frequency use of the 59 tested quality ques showed that at the sample level, freshness and taste were among the most frequently used to assess food quality (Table 4). A statistically significant difference between countries was observed for most cues: 78% of them. In 91.3% of the cases with a difference, a higher mean rank of scores was found among the Romanian consumers (Table 4). 

In many cases, the scores assigned by the Romanian group to the use frequency of cues for food quality evaluation were higher than the ones given by Belgians. This difference was probably the consequence of an overall higher appreciation of the cues involved in food quality evaluation and of higher attention given to the food quality assessment process. The latter assumption seemed to be supported by consumers’ answers to the question about attention assigned to food quality (higher for Romanians). However, it should be considered that other factors besides interest (attention) intervene in explaining the use frequency of quality cues, similarly to the case of the intention–behavior gap for various food related behaviors [117,118].

Consumer’s sources of information are generally grouped into internal versus and external sources [119] and personal versus impersonal sources [120,121]. External sources of information represent information from sources other than internal memory (objective and subjective knowledge), such as friends, family, packages, and sales personnel. The confidence in a source changes according to the object about which the source communicates something. Thus, for functional food, the sources in which respondents have most confidence are doctors and public entities, trusted by 42% and 39% of consumers, respectively, while a lesser degree of confidence is given to producers (trusted by 32% of consumers) and product labels (34%) [122]. Doctors and research institutes were also the most trusted sources of information for several possible food related hazards: additives, GMOs, residues, nutritionally imbalanced food [123]. In the case of the present study, consumers had the highest confidence level in their judgement (Table 5) regarding the power to obtain accurate information about the quality of the food they buy. This level was statistically significant higher compared to the one assigned to all other tested information sources (based on the results of the Wilcoxon signed ranks test). 

The Mann–Whitney U test revealed that the existence of health problems (for the consumer or his/her family) did not generate a statistically significant difference in the use frequency of the food quality cues.

The number of cues that consumers most frequently used to assess food healthiness covered 70% of the maximum number of answers (when all 797 respondents would have named five cues). In the first case, the cues mentioned in the open ended question were aggregated into 41 groups, detailed in Table 6. The three most important cues that conveyed information about the healthiness of food were ingredients, nutrition facts, and additives (Table 6). 

Many factors can influence the healthy/unhealthy categorization of foods, such as ingredients, fat content [124], nutrition facts, and calories. To evaluate food healthiness, consumers in this study especially used ingredients, nutrition facts, and additives (Table 6). These results were partly in line with a study performed in the USA (using low income, predominantly black community participants), which showed that healthy and unhealthy food was especially defined by food additives, nutrient content, food packaging, and production and processing methods [125]. Furthermore, a study using Swiss participants showed that perceived healthiness was especially determined by fat content, organic label, and processing [126]. Paquette [127] wisely observed that the perception of healthy eating was one of the many determinants of eating patterns.

Knowledge of how consumers evaluate food healthiness is an important piece of information for any education campaign aimed at improving eating habits in order to make them healthier. At the same time, it can be observed that the same cues are also frequently used for food quality assessment (Table 4), stressing again the importance of making them available for consumers.

Consumer perceived control of the capacity to influence health towards one of the three possible directions, maintain, improve, or damage, was high in all cases (Table 7). The high strength of this belief is a good foundation on which marketing action can rely in order to encourage a shift towards healthier eating habits or to reinforce current good ones. 

Standard linear regression was performed four times in order to observe how well six independent variables (1. attention paid to food quality, 2. confidence in self-capacity to evaluate food quality, 3. perceived control to maintain, 4. improve, and 5. damage health through food choices, and 6. BMI) could predict the use frequency of several food quality cues related to health (dependent variable). The tested cues were: ingredient list, fat content, salt content, and sugar content (Table 8).

**Figure 1:**
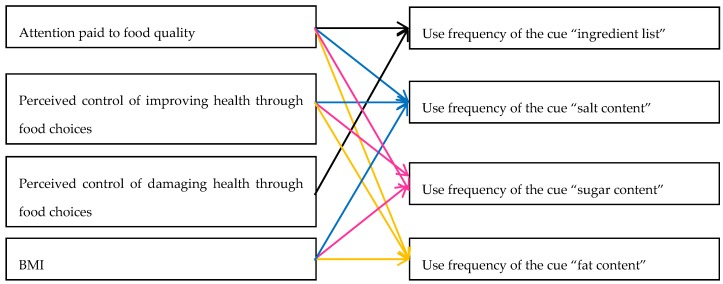
Model of observed determinants of the use frequency of food quality cues related to health, based on the results of standard linear regression.

The use frequency of all tested health cues was influenced to the strongest degree by the attention paid to food quality. In the case of salt, sugar, and fat content, the influence of the perceived control to improve health by eating the right foods and of the BMI was also present. Regarding the use frequency of ingredient list, it was observed that the belief that he/she could damage his/her health by eating the wrong foods influenced the use frequency of this cue (Figure 1). It could be concluded that attention to and perceived control of damaging health by eating the wrong foods explained 14.9% of the variance in the use frequency of ingredient list, that attention to and perceived control of improving health by eating the right foods, and BMI explained together 13.2%, 11.3%, and 11.1%, respectively, of the variance in the use frequency of salt content, sugar content, and fat content, respectively (Table 8). Even if these percentages were not high, they could be considered acceptable in the context of the present research due to the fact that it is rather difficult to discover variables that can explain the use frequency of food quality cues and to the fact that this is the first study (to the authors’ best knowledge) that makes a prediction regarding which variables have an influence on the use frequency of food quality cues [27].

Environmental concern is gaining importance in consumer food choices, generating changes in production and supply and thus demonstrating how the consumer can contribute to environmental health.

In this study, the three most important cues used for the evaluation of the environmental impact of food were packaging, origin, and production type. This was not in line with research showing that 85 Swiss participants indicated that organic labels and provenance were among the main predictors of the products’ perceived environmental friendliness [125]. In the present study, packaging was by far the most relevant indicator of food environmental impact for consumers surveyed in this study. In other words, perception of environment impact depends on packaging. Even if, according to food type, the real main environmental impact may be different, such as water consumption [127] or deforestation for beef meat production, packaging is for sure a very easy to use indicator for consumers, being visible at the moment of purchase and easy to evaluate from the point of view of material type and quantity. Given the resource consumption and pollution generated along the entire life cycle of packaging products, this belief can be successfully used in marketing campaigns to stimulate consumers to choose sustainable packaging options, such as products with less packaging (e.g., bulk products), recyclable packaging, and packaging made of recycled or other environmentally friendly materials.

The number of cues that consumers most frequently took into account to assess food impact on the environment represented 50.3% of the maximum number of answers. These cues (mentioned in an open ended question) were aggregated into 45 groups, detailed in Table 9, and the most important of them were packaging, origin, and production type (Table 9).

From a practical perspective, this investigation could provide useful information for the study of the intention to buy a food product by revealing the cues that are the most frequently used by consumers, with implications on consumer health and a cleaner environment. The presence of health and environmental cues can support the efforts of stakeholders interested in these directions to increase the efficiency of their actions that target food consumption behavior. For example, the information can be used in education-information [27] campaigns about the effects of specific nutrients to safeguard health or about the environmental impact of some foods to stimulate choices that support environmental health, for example through lower pollution. 

Summing up, this paper highlighted some important cues in Romanian and Belgian consumers’ understanding of food quality referring to food in general and not to a specific food product. This is critical for marketing researchers and practitioners to define marketing programs fitting the increasing high quality food demand. 

The results of the present study should be regarded in the context of their limitations. The number of potential determinants of the use frequency of food quality cues can be increased in future research in order to discover more about this relationship. Furthermore, the investigation of consumer understanding of food healthiness and environmental impact can be extended through the use of other variables such as level of healthiness, environmental damage, and water footprint. The understanding of freshness should be clarified, as it can embed attributes that differ among consumers. The sample was not statistically representative at the country level, and a future study could fill in this gap. The present study focused on the perception of quality in general; therefore, some product characteristics such as freshness or fair trade certification may have high relevance for specific foods and low for others. Future research could consider investigating whether and to what extent different quality cues differ among specific types of products. Additional statistical analyses should be run in future research in order to identify segments of consumers according to the food quality cues that they use. Despite these limitations, the results of this study can contribute to the efforts of informing and educating consumers to adopt healthier and more environmentally friendly food choices.

## 5. Conclusions

Much depends on consumers’ food quality perception: profits, health, and nature. The economic benefits resulting from food purchasing acts are contributory toward consumers’ quality evaluation. Perception of food quality influences dietary patterns with implicit consequences on health both at the individual and society level. The environmental impact of the entire food chain, from farm to garbage bin, is influenced by consumers’ choices.

The present study placed a strong emphasis on food quality cues related to health and environmental protection. The reason why we proceeded in this way was on the one hand that it is widely accepted that health and environmental concerns are important factors in consumer decisions [128]. On the other hand, these credence attributes (health and environment) pose a significant challenge to consumers’ quality evaluation as they cannot determine the product’s quality even after the purchase or consuming act. In the process of undertaking this challenge, a richness of concepts dedicated to quality cues was encountered, and they were incorporated in the analysis. Consistent behavioral differences have been observed in the food choices area in general [129], and in particular in food quality perception, as food quality is a multi-dimensional concept influenced by contextual factors. We assumed that quality evaluation is a heterogeneous act, and this implied a context specific examination. We focused on perceptions as key issue of every marketing strategy targeting food quality is consumers’ perceptions of quality. Consumers’ quality evaluation is a subjective process attributed to their characteristics, such as demographics and cultural background, and therefore, it is expected to have differences from one country to another or between age or income groups. 

This contribution highlighted the importance of food quality evaluation for health and environment protection, and it developed a framework for the understanding of food quality evaluation based on the investigation of Romanian and Belgian food consumers. The results emphasized that the investigated consumers assigned high attention to food quality and used freshness, taste, and appearance the most frequently to evaluate food quality. The use frequency of food quality cues related to health was primarily influenced by the attention paid to food quality. The packaging was the main indicator of the environmental impact of food, thus highlighting a possible intervention for less pollution and resource consumption related to packaging, which can be more easily accepted by consumers.

Environmental protection, healthy diets, and human progress are inter-dependent, and that is why building a sustainable food system that prioritizes consumers’ needs and preferences can support the shift towards a sustainable consumption paradigm. As long as quality is a central concept in food choices, the insights into consumers’ understanding of food quality is one of the building blocks that lays the foundation of a sustainable consumption pattern. 

## Figures and Tables

**Table 1:** Quality cues used in the present investigation and some of the studies where they were previously included.

	Quality Cues:	Studies that Used the Cue		Quality Cues:	Studies that Used the Cue
1.	Price ((Price))	[52,53]; FG*	30.	Familiarity for you (the fact that you know the product well) ((Familiarity))	[54,55,56,57]; FG
2.	Appearance ((Appearance))	[58,59,60]; FG	31.	Country of origin ((Country of origin))	[61,62]; FG
3.	Smell ((Smell))	[63,64]; FG	32.	Name of producer ((Producer name))	FG
4.	Taste (Taste)	[54,65]; FG	33.	Name of importer (Importer name)	[55]; FG
5.	Quantity sold (Quantity)	[66,67]; FG	34.	Brand (Brand)	[1,53]; FG
6.	Availability: being easy to find on the market (Availability)	[53]	35.	Being a product for children (Product for children)	[68]; FG
7.	Expiration date: best before/use by date (Expiration date)	FG	36.	Being a product for diabetics (Product for diabetics)	[69]; FG
8.	Ingredient list (Ingredients)	[70,71,72]; FG	37.	Being a natural product (Natural product)	[73,74]; FG
9.	Fat content (Fat)	[71,72]; FG	38.	Being a traditional product (Traditional product)	[75]; FG
10.	Salt content (Salt)	[71,72]; FG	39.	Being a local product (Local product)	[56,76,77,78]; FG
11.	Sugar content (Sugar)	[71,72]; FG	40.	Being made in the EU (EU product)	[77]; FG
12.	Calories (Calories)	[71,72]; FG	41.	Being a product from the mountains (Product from the mountain)	[75,78]; FG
13.	Vitamins and minerals (Vitamins, Minerals)	[71]; FG	42.	Being a free-range product (animals can roam freely outdoors, at least part of the day) (Free-range product)	[79,80]; FG
14.	Fibers (Fibers)	[71]; FG	43.	Being a product made of wild animals or plants (Product made of wild animals or plants)	FG
15.	Proteins (Proteins)	[71]; FG	44.	Certification label: organic product (Organic product)	[81,82]; FG
16.	Coloring agents (Content: coloring)	[71]; FG	45.	Certification label: ISO (ISO)	[82]; FG
17.	Preservatives (Content: preservatives)	[83]; FG	46.	Certification label: Protected designation of origin, protected geographical indication, traditional specialties guaranteed (PDO, PGI, TSG)	[83]
18.	Taste enhancers (Content: Taste enhancers)	[84]; FG	47.	Certification label: fair trade (Fair Trade)	[82,83]
19.	Other chemical additives (Content: Other additives)	[72]; FG	48.	Certification label: Rainforest Alliance, Carbon Footprint (Rainforest Alliance, CO2 Footprint)	[57,85,86,87]; FG
20.	Genetically modified organisms (GMOs) (Content: GMOs)	[88,89,90]; FG	49.	Social equity information: about the respect of community and producers’ rights, about the use/non-use of child labor in production of the evaluated food, of poor working conditions and wages for food producers, the contribution to reducing starvation and malnutrition or improving the life of people in need (Social equity)	[57]; FG
21.	Cloned animals (Content: Cloned animals)	[91]; FG	50.	Information about deforestation/reforestation (e.g., soy was not/ was produced in fields created though deforestation of the rain forest; x% of the price is used for reforestation) (Deforestation, reforestation)	[57,86]; FG
22.	Allergens (Allergens)	[71,84]; FG	51.	Information about the use of chemical/natural fertilizers and pesticides used in production of the evaluated food (Fertilizers, pesticides)	[57]; FG
23.	Type of processing: fried, dried, raw, etc. (Processing type)	[62,70]; FG	52.	Information about the pollution generated by production of the evaluated food (toxic emissions, carbon emissions, etc.) (Pollution)	[57]; FG
24.	Hygiene standards: how it is processed, handled, stored, displayed (Hygiene)	[92,93]; FG	53.	Information about the treatment of animals in the production of the evaluated food: animal welfare (if they lived in cages or not, etc.) (Animal welfare)	[85,87]; FG
25.	Easiness to prepare (Easy preparation)	[55]; FG	54.	Information about the use of resources in the production or transportation of the evaluated food (water, energy, or other) (Resources used)	[85,94]; FG
26.	Cooking instructions (Cooking instructions)	[56]; FG	55.	Information about the recyclable character of packaging and about the amount of packaging used on products (Recyclable and amount of packaging)	[73]; FG
27.	Storing instructions (Storing instructions)	[95]; FG	56.	Information about the loss of biodiversity caused by production of the evaluated food (extinction or reduction in animal/plant populations due to pollution, replacement of traditional/endemic species with commercial ones, etc.) (Loss of biodiversity)	[96,97]; FG
28.	The fact that it is new on the market (New on the market)	[98]; FG	57.	Information about the amount of waste generated through production, transportation, storage, and consumption of the evaluated food (Generated waste)	[99]; FG
29.	The fact that many people eat it, its popularity (Many eat it)	[56,100]; FG	58.	Freshness (Freshness)	[1,78]; FG
			59.	Packaging material (paper, PET, plastic, etc.) (Packaging material)	[101,102]; FG

* Mentioned during the focus group (FG); ** The short name between square brackets is used hereinafter for this cue.

**Table 2:** Summary statistics (N = 797).

Variable	Description	Frequency (% in Total Sample)	Mean (Total Sample)
Gender	Men	36	
	Women	64	
BMI	BMI score		22.6
Health status	The respondent		
	Health problem	7	
	No health problems	93	
	The respondent’s family members		
	Health problem	31	
	No health problems	69	
	Not the respondent, nor his/her family	65	
	Both the respondent and his/her family	4	
Living environment	Urban	51	
	Rural	49	
Age			26
Nationality	Belgian	55	
	Romanian	45	

**Table 3:** Percentage of tested consumers who declared they use often a specific cue for the evaluation of food quality (unaided awareness).

Cues	Frequency (% in Total Sample)	Cues	Frequency (% in Total Sample)	Cues	Frequency (% in Total Sample)
appearance (it includes: appearance, color, size, and shape)	52.7	producer	8.3	selling place	2.3
price	38.3	additives	7.5	calories	2.1
ingredients	31.0	seasonal product	6.5	variety	2.1
origin (country)	27.9	texture	6.5	consumers’ opinion	1.9
taste	27.0	processing level (raw, pre-cooked, cooked, etc.)	5.8	storage conditions	1.3
freshness	26.5	ethical aspects	4.3	hygiene	1.3
organic label	26.2	production type (small scale, intensive, etc.)	4.3	pesticides	1.1
expiration date	21.2	healthiness	4.1	food type (vegetal, animal)	1.0
smell	19.7	quantity	3.5	previous experience with the product	1.0
local product	14.1	processing type (fried, boiled, etc.)	3.1	seller advice	0.8
packaging	13.9	environmental impact	2.6	GMOs	0.5
brand	12.8	natural character	2.6	cooking instructions	0.4
nutrition facts (except for calories)	10.4	quality labels	2.5	availability	0.4

**Table 4:** Use frequency of quality cues by tested consumers (average scores on a scale from 1 = extremely rarely to 7 = extremely often; cues are listed in the table from the most frequently used one to the least frequently used one based on the results for the total sample).

Cue	T *	B *	R *	Cue	T	B	R	Cue	T	B	R
Freshness	5.7	5.8	5.6	Brand	4.4	4.1	4.6	Easy preparation	3.8	3.3	4.4
Taste	5.6	5.4	5.8 **	Content: GMOs	4.3	4.1	4.5	Rainforest, CO2 footprint	3.8	3.8	3.8
Appearance	5.5	5.3	5.7	Fat	4.2	3.8	4.8	Producer name	3.8	3.3	4.4
Price	5.3	5.4	5.3	Traditional product	4.2	3.9	4.6	Deforestation, reforestation	3.7	3.6	3.8
Smell	5.3	4.9	5.7	Fair Trade	4.1	4.4	3.8	ISO	3.7	3.4	4.2
Ingredients	5.3	5.1	5.5	PDO, PGI, TSG	4.1	4.1	4.2	Social equity	3.7	3.5	3.9
Expiration date	5.2	4.8	5.7	Availability	4.2	3.7	4.7	Storing instructions	3.6	3.2	4.2
Familiarity	5.2	5.0	5.4	Vitamins, Minerals	4.1	3.5	4.9	Allergens	3.6	3.1	4.2
Local product	5.0	5.3	4.6	Proteins	4.1	3.5	4.8	Pollution	3.5	3.3	3.8
Packaging material	4.9	5.0	4.8	Processing type	4.1	3.7	4.6	Loss of biodiversity	3.5	3.4	3.7
Organic product	4.9	5.1	4.7	Animal welfare	4.1	4.1	3.7	Generated waste	3.5	3.3	3.8
Natural product	4.9	4.7	5.1	EU product	4.1	4.0	4.2	Product from the mountain	3.5	2.9	4.3
Country of origin	4.8	5.2	4.3	Calories	4.1	3.6	4.7	Resources used	3.5	3.2	3.8
Quantity	4.7	4.7	4.7	Fibers	4.1	3.5	4.7	Cooking instructions	3.3	2.6	4.2
Free-range product	4.5	4.4	4.6	Salt	4.0	3.6	4.5	Many eat it	3.2	2.5	4.2
Content: Other additives	4.5	4.2	4.7	Hygiene	4.0	3.4	4.9	New on the market	3.2	2.6	3.9
Content: Preservatives	4.4	4.1	4.7	Fertilizers, pesticides	3.9	3.8	4.1	Importer name	3.1	2.6	3.7
Content: Coloring	4.4	4.0	4.8	Recyclable and amount of packaging	3.9	3.9	4.0	Product for children	2.9	2.0	4.0
Content: Taste enhancers	4.4	4.1	4.6	Content: Cloned animals	3.9	3.6	4.2	Product for diabetics	2.8	1.8	3.9
Sugar	4.4	4.0	4.8	Product made of wild animals, plants	3.9	3.5	4.3				

* T = total sample (Belgian and Romanian consumers); B = Belgian sample; R = Romanian sample; ** The underline indicates the country with a higher mean rank in the situation when a statistically significant difference between scores was revealed by the Mann-Whitney U test.

**Table 5:** Consumers’ average confidence level in the accuracy of information regarding food quality.

Producers of the Food, through Their Website	Producers of the Food, through the Information on the Label and Package	Sellers/Shopping Assistants in the Shops where the Consumer Buys the Food	Consumer’s Family and Friends	Other Consumers	Consumer’s Own Judgement
3.6	4.2	3.6	5.0	4.1	5.4

**Table 6:** Percentage of tested consumers who declared they often used a specific cue for the evaluation of food healthiness (unaided awareness).

Cues	Frequency (% in Sample of 797 Consumers)	Cues	Frequency (% in Sample of 797 Consumers)	Cues	Frequency (% in Sample of 797 Consumers)
ingredients	25.3	processing type	8.3	hygiene standards	1.5
nutrition facts (salt, sugar, fat, other)	29.7	packaging	7.5	food type	1.1
additives	28.2	ethical	7.4	storage conditions	1.0
freshness	24.0	production type	6.5	consumers’ opinion	0.9
organic label	23.7	producer	6.4	nutrition experts’ opinion	0.8
origin	23.3	price	5.8	friends’ recommendations	0.6
appearance	20.3	natural	5.4	coloring agents	0.4
expiration date	14.4	color	3.6	preservatives	0.4
smell	11.4	quality labels	3.6	previous experience with the product	0.4
taste	10.9	seasonal product	3.6	quantity	0.3
local product	10.4	environmental impact	3.3	selling place	0.3
calories	8.5	GMOs	3.3	cooking instructions	0.1
processing level	8.4	brand	2.3	distributor	0.1
pesticides	8.3	texture	1.9		

**Table 7:** Perceived control of the capacity to influence health towards one of the three possible directions: maintain, improve, damage (average scores at total sample level).

Capacity to Maintain Current Health State by Eating the Right Foods	Capacity to Improve Current Health State by Eating the Right Foods	Capacity to Damage Current Health State by Eating the Wrong Foods
5.9	5.7	5.7

**Table 8:** Results of standard linear regression tests.

Independent Variable	Dependent Variable	Standardized Coefficients (beta)	Unstandardized Coefficients (B)	Standard Error (SE)	*p* for Independent Variables	R Squared	*p* for the Model
*Attention*	Use frequency: Ingredient list	0.284	0.455	0.055	*0.000*	0.149	0.000
Self-confidence		0.013	0.019	0.049	0.695		
Perceived control: maintain		0.070	0.105	0.066	0.113		
Perceived control: improve		0.081	0.106	0.056	0.058		
*Perceived control: damage*		0104	0.110	0.037	*0.003*		
BMI		−0.035	−0.016	0.016	0.296		
							
*Attention*	Use frequency: Salt content	0.261	0.489	0.065	*0.000*	0.132	0.000
Self-confidence		−0.017	−0.028	0.058	0.625		
Perceived control: maintain		−0.033	−0.058	0.078	0.462		
*Perceived control: improve*		0.210	0.324	0.066	*0.000*		
Perceived control: damage		0.037	0.046	0.044	0.290		
*BMI*		0.073	0.040	0.018	*0.029*		
*Attention*	Use frequency: Sugar content	0.223	0.416	0.065	*0.000*	0.113	0.000
Self-confidence		−0.036	−0.059	0.058	0.308		
Perceived control: maintain		0.004	0.007	0.079	0.929		
*Perceived control: improve*		0.185	0.283	0.067	*0.000*		
Perceived control: damage		0.042	0.052	0.044	0.239		
*BMI*		0.095	0.052	0.019	*0.005*		
*Attention*	Use frequency: Fat content	0.226	0.416	0.064	*0.000*	0.111	0.000
Self-confidence		−0.032	−0.052	0.057	0.360		
Perceived control: maintain		−0.033	−0.057	0.078	0.464		
*Perceived control: improve*		0.196	0.297	0.066	*0.000*		
Perceived control: damage		0.057	0.069	0.043	0.111		
*BMI*		0.117	0.063	0.018	*0.001*		

**Table 9:** Percentage of tested consumers who declared they use often a specific cue for the evaluation of food impact on the natural environment (unaided awareness).

Cues	Frequency (% in Sample of 797 Consumers)	Cues	Frequency (% in Sample of 797 Consumers)	Cues	Frequency (% in Sample of 797 Consumers)
packaging	38.5	recycling	4.8	energy consumption	0.8
origin	27.1	seasonal product	4.8	expiration date	0.6
production type	21.1	biodiversity loss	4.6	natural	0.6
pesticides	15.8	biodegradable	4.5	quantity	0.6
ethical	13.4	food type	3.8	appearance	0.5
organic label	11.9	processing type	3.8	experts’ opinion	0.5
local product	10.8	additives	3.5	freshness	0.5
generated waste	8.7	CO2 emissions	3.1	animal treatment	0.4
ingredients	8.7	brand	2.9	cooking instructions	0.4
transport	8.3	GMOs	2.4	color	0.1
resources used	7.9	plastic quantity	2.4	friends’ recommendations	0.1
pollution	7.8	producer	1.5	quality labels	0.1
deforestation	7.7	storage conditions	1.5	nutrition facts	0.1
general environmental impact	6.4	price	1.4	selling place	0.1
quality labels	5.9	processing level	1.3	taste	0.1

## References

[1] Mascarello G., Pinto A., Parise N., Crovato S., Ravarotto L. (2015). The perception of food quality. Profiling Italian consumers. Appetite.

[2] Sajdakowska M., Gębski J., Gutkowska K., Żakowska-Biemans S. (2018). Importance of Health Aspects in Polish Consumer Choices of Dairy Products. Nutrients.

[3] Grunert K.G. (2005). Food quality and safety: Consumer perception and demand. Eur. Rev. Agric. Econ..

[4] Baiardi D., Puglisi R., Scabrosetti S. (2016). Individual attitudes on food quality and safety: Empirical evidence on EU countries. Food Qual. Prefer..

[5] Van Rijswijk W., Frewer L.J. (2008). Consumer perceptions of food quality and safety and their relation to traceability. Br. Food J..

[6] Gilmore S., Brown N., Dana J. (1998). Food quality model for school foodservices. J. Child Nutr. Manag..

[7] Van Ooijen I., Fransen M.L., Verlegh P.W.J., Smit E.G. (2017). Packaging design as an implicit communicator: Effects on product quality inferences in the presence of explicit quality cues. Food Qual. Prefer..

[8] De Almeida M., Graca P., Lappalainen R., Giachetti I., Kafatos A., Remaut de Winter A., Kearney J. (1997). Sources used and trusted by nationally-representative adults in the European Union for information on healthy eating. Eur. J. Clin. Nutr..

[9] Magnier L., Schoormans J., Mugge R. (2016). Judging a product by its cover: Packaging sustainability and perceptions of quality in food products. Food Qual. Prefer..

[10] Verbeke W., Ward R.W. (2006). Consumer interest in information cues denoting quality, traceability and origin: An application of ordered probit models to beef labels. Food Qual. Prefer..

[11] Kolodinsky J. (2012). Persistence of health labeling information asymmetry in the United States: Historical perspectives and twenty-first century realities. J. Macromark..

[12] Petrescu D.-C., Orolan I.G., Proorocu M., Mihăiescu T., Paulette L., Vârban D. (2013). Organic products: Consumption habits and perceptions. Adv. Environ. Sci..

[13] Petrescu-Mag R., Petrescu D., Sima N.-F., Sima R. (2016). Informed product choice in the organic food sector: From guaranteeing the legal rights to facing sustainability challenges. J. Environ. Prot. Ecol..

[14] Cerri J., Testa F., Rizzi F. (2018). The more I care, the less I will listen to you: How information, environmental concern and ethical production influence consumers’ attitudes and the purchasing of sustainable products. J. Clean. Prod..

[15] Festila A., Chrysochou P. (2018). Implicit communication of food product healthfulness through package design: A content analysis. J. Consum. Behav..

[16] Osburg V.-S., Yoganathan V., Brueckner S., Toporowski W. (2019). How detailed product information strengthens eco-friendly consumption. Manag. Decis..

[17] Atkinson L., Rosenthal S. (2014). Signaling the green sell: The influence of eco-label source, argument specificity, and product involvement on consumer trust. J. Advert..

[18] Connelly B.L., Certo S.T., Ireland R.D., Reutzel C.R. (2011). Signaling theory: A review and assessment. J. Manag..

[19] Krystallis A., Fotopoulos C., Zotos Y. (2006). Organic consumers’ profile and their willingness to pay (WTP) for selected organic food products in Greece. J. Int. Consum. Mark..

[20] Prentice C., Chen J., Wang X. (2019). The influence of product and personal attributes on organic food marketing. J. Retail. Consum. Serv..

[21] Ditlevsen K., Sandøe P., Lassen J. (2019). Healthy food is nutritious, but organic food is healthy because it is pure: The negotiation of healthy food choices by Danish consumers of organic food. Food Qual. Prefer..

[22] Brunsø K., Fjord T.A., Grunert K.G. (2002). Consumers’ Food Choice and Quality Perception.

[23] Aschemann-Witzel J., Peschel A.O. (2019). How circular will you eat? The sustainability challenge in food and consumer reaction to either waste-to-value or yet underused novel ingredients in food. Food Qual. Prefer..

[24] Van Loo E.J., Hoefkens C., Verbeke W. (2017). Healthy, sustainable and plant-based eating: Perceived (mis) match and involvement-based consumer segments as targets for future policy. Food Policy.

[25] Brecic R., Mesic Z., Cerjak M. (2017). Importance of intrinsic and extrinsic quality food characteristics by different consumer segments. Br. Food J..

[26] Januszewska R., Pieniak Z., Verbeke W. (2011). Food choice questionnaire revisited in four countries. Does it still measure the same?. Appetite.

[27] Lancaster K. (1971). Consumer Demand: A New Approach.

[28] Kramer A., Twigg B. (1968). Measure of frozen food quality and quality changes. The Freezing Preservation of Foods.

[29] Molnar P. (1995). A model for overall description of food quality. Food Qual. Prefer..

[30] Zeithaml V.A. (1988). Consumer perceptions of price, quality, and value: A means-end model and synthesis of evidence. J. Mark..

[31] Saba A., Sinesio F., Moneta E., Dinnella C., Laureati M., Torri L., Peparaio M., Civitelli E.S., Endrizzi I., Gasperi F. (2019). Measuring consumers attitudes towards health and taste and their association with food-related life-styles and preferences. Food Qual. Prefer..

[32] McInemey J. (2002). The production of food: From quantity to quality. Proc. Nutr. Soc..

[33] Petrescu-Mag R. (2011). Protecţia Mediului în Contextul Dezvoltării Durabile (Environmental Protection in the Context of Sustainable Development. Legislations and Institutions).

[34] Yu H., Gibson K.E., Wright K.G., Neal J.A., Sirsat S.A. (2017). Food safety and food quality perceptions of farmers’ market consumers in the United States. Food Control.

[35] Caswell J.A., Mojduszka E.M. (1996). Using informational labeling to influence the market for quality in food products. Am. J. Agric. Econ..

[36] Hooker N.H., Caswell J.A., Caswell J.A. (1996). Regulatory Targets and Regimes for Food Safety: A Comparison of North American and European Approaches. The Economics of Reducing Health Risk from Food.

[37] Han J., Ruiz-Garcia L., Qian J., Yang X. (2018). Food packaging: A comprehensive review and future trends. Compr. Rev. Food Sci. Food Saf..

[38] Kim K.-H., Lee K.-R. (2019). What Are South Korean Consumers’ Concerns When Buying Eco-Friendly Agricultural Products?. Sustainability.

[39] Verbeke W., Pérez-Cueto F.J., de Barcellos M.D., Krystallis A., Grunert K.G. (2010). European citizen and consumer attitudes and preferences regarding beef and pork. Meat Sci..

[40] Nelson P. (1970). Information and consumer behavior. J. Political Econ..

[41] Nelson P. (1974). Advertising as information. J. Political Econ..

[42] Darby M.R., Karni E. (1973). Free competition and the optimal amount of fraud. J. Law Econ..

[43] Ford G.T., Smith D.B., Swasy J.L. (1988). An empirical test of the search, experience and credence attributes framework. Adv. Consum. Res..

[44] Olson J.C., Jacoby J. (1972). Cue Utilization in the Quality Perception Process; in SV - Proceedings of the Third Annual Conference of the Association for Consumer Research. https://www.acrwebsite.org/search/view-conference-proceedings.aspx?Id=11997.

[45] Chung J.-E., Pil Yu J., Thorndike Pysarchik D. (2006). Cue utilization to assess food product quality: A comparison of consumers and retailers in India. Int. Rev. Retai. Distrib. Consum. Res..

[46] Caputo V., Scarpa R., Nayga R.M., Ortega D.L. (2018). Are preferences for food quality attributes really normally distributed? An analysis using flexible mixing distributions. J. Choice Model..

[47] Steenkamp J.-B.E.M. (1990). Conceptual model of the quality perception process X. J. Bus. Res..

[48] Grunert K.G., Larsen H.H., Madsen T.K., Baadsgaard A. (1996). Market Orientation in Food and Agriculture.

[49] Bredahl L. (2004). Cue utilisation and quality perception with regard to branded beef. Food Qual. Prefer..

[50] Chamhuri N., Batt P.J. (2015). Consumer perceptions of food quality in Malaysia. Br. Food J..

[51] Choi Y., Lee J. (2019). The effect of extrinsic cues on consumer perception: A study using milk tea products. Food Qual. Prefer..

[52] Ryu K., Han H. (2010). Influence of the quality of food, service, and physical environment on customer satisfaction and behavioral intention in quick-casual restaurants: Moderating role of perceived price. J. Hosp. Tour. Res..

[53] Wang X., Li D. (2012). A dynamic product quality evaluation based pricing model for perishable food supply chains. Omega.

[54] Almli V.L., Verbeke W., Vanhonacker F., Næs T., Hersleth M. (2011). General image and attribute perceptions of traditional food in six European countries. Food Qual. Prefer..

[55] Santosa M., Abdi H., Guinard J.-X. (2010). A modified sorting task to investigate consumer perceptions of extra virgin olive oils. Food Qual. Prefer..

[56] Sirieix L., Delanchy M., Remaud H., Zepeda L., Gurviez P. (2013). Consumers’ perceptions of individual and combined sustainable food labels: A UK pilot investigation. Int. J. Consum. Stud..

[57] Verbeke W., Vermeir I., Brunsø K. (2007). Consumer evaluation of fish quality as basis for fish market segmentation. Food Qual. Prefer..

[58] Barrett D.M., Beaulieu J.C., Shewfelt R. (2010). Color, flavor, texture, and nutritional quality of fresh-cut fruits and vegetables: Desirable levels, instrumental and sensory measurement, and the effects of processing. Crit. Rev. Food Sci. Nutr..

[59] Cheng J., Sun D., Han Z., Zeng X. (2014). Texture and structure measurements and analyses for evaluation of fish and fillet freshness quality: A review. Compr. Rev. Food Sci. Food Saf..

[60] Wu D., Sun D.-W. (2013). Colour measurements by computer vision for food quality control—A review. Trends Food Sci. Technol..

[61] Claret A., Guerrero L., Aguirre E., Rincón L., Hernández M.D., Martínez I., Peleteiro J.B., Grau A., Rodríguez-Rodríguez C. (2012). Consumer preferences for sea fish using conjoint analysis: Exploratory study of the importance of country of origin, obtaining method, storage conditions and purchasing price. Food Qual. Prefer..

[62] Pouta E., Heikkilä J., Forsman-Hugg S., Isoniemi M., Mäkelä J. (2010). Consumer choice of broiler meat: The effects of country of origin and production methods. Food Qual. Prefer..

[63] Manzocco L., Rumignani A., Lagazio C. (2013). Emotional response to fruit salads with different visual quality. Food Qual. Prefer..

[64] Simion A.M.C., Vizireanu C., Alexe P., Franco I., Carballo J. (2014). Effect of the use of selected starter cultures on some quality, safety and sensorial properties of Dacia sausage, a traditional Romanian dry-sausage variety. Food Control.

[65] Stewart P.C., Goss E. (2013). Plate shape and colour interact to influence taste and quality judgments. Flavour.

[66] Dörnyei K.R., Krystallis A., Chrysochou P. (2017). The impact of product assortment size and attribute quantity on information searches. J. Consum. Mark..

[67] Wilson N.L.W., Rickard B.J., Saputo R., Ho S.-T. (2017). Food waste: The role of date labels, package size, and product category. Food Qual. Prefer..

[68] Petrescu D.C., Petrescu-Mag R.M., Burny P., Azadi H. (2017). A new wave in Romania: Organic food. Consumers’ motivations, perceptions, and habits. Agroecol. Sustain. Food Syst..

[69] Dean M., Lampila P., Shepherd R., Arvola A., Saba A., Vassallo M., Claupein E., Winkelmann M., Lähteenmäki L. (2012). Perceived relevance and foods with health-related claims. Food Qual. Prefer..

[70] Fernqvist F., Ekelund L. (2014). Credence and the effect on consumer liking of food—A review. Food Qual. Prefer..

[71] Hoefkens C., Verbeke W., Van Camp J. (2011). European consumers’ perceived importance of qualifying and disqualifying nutrients in food choices. Food Qual. Prefer..

[72] Lähteenmäki L., Lampila P., Grunert K., Boztug Y., Ueland Ø., Åström A., Martinsdóttir E. (2010). Impact of health-related claims on the perception of other product attributes. Food Policy.

[73] Milošević J., Žeželj I., Gorton M., Barjolle D. (2012). Understanding the motives for food choice in Western Balkan Countries. Appetite.

[74] Petrescu D.C., Petrescu-Mag R.M., Burny P. (2015). Management of environmental security through organic agriculture. Contribution of consumer behavior. Environ. Eng. Manag. J..

[75] Cerjak M., Haas R., Brunner F., Tomić M. (2014). What motivates consumers to buy traditional food products? Evidence from Croatia and Austria using word association and laddering interviews. Br. Food J..

[76] Arsil P., Li E., Bruwer J., Lyons G. (2014). Exploring consumer motivations towards buying local fresh food products: A means-end chain approach. Br. Food J..

[77] Bojnec S., Petrescu D.C., Petrescu-Mag R.M., Radulescu C.V. (2019). Locally Produced Organic Food: Consumer Preferences. Amfiteatru Econ..

[78] Sidali K.L., Kastenholz E., Bianchi R. (2015). Food tourism, niche markets and products in rural tourism: Combining the intimacy model and the experience economy as a rural development strategy. J. Sustain. Tour..

[79] García-Torres S., López-Gajardo A., Mesías F. (2016). Intensive vs. free-range organic beef. A preference study through consumer liking and conjoint analysis. Meat Sci..

[80] Michaelidou N., Hassan L.M. (2010). Modeling the factors affecting rural consumers’ purchase of organic and free-range produce: A case study of consumers’ from the Island of Arran in Scotland, UK. Food Policy.

[81] Howard P.H., Allen P. (2010). Beyond organic and fair trade? An analysis of ecolabel preferences in the United States. Rural Sociol..

[82] Zander K., Hamm U. (2010). Consumer preferences for additional ethical attributes of organic food. Food Qual. Prefer..

[83] Aprile M.C., Caputo V., Nayga R.M. (2012). Consumers’ valuation of food quality labels: The case of the European geographic indication and organic farming labels. Int. J. Consum. Stud..

[84] Lähteenmäki L. (2013). Claiming health in food products. Food Qual. Prefer..

[85] Banterle A., Cereda E., Fritz M. (2013). Labelling and sustainability in food supply networks: A comparison between the German and Italian markets. Br. Food J..

[86] De Andrade Silva A.R., Bioto A.S., Efraim P., de Castilho Queiroz G. (2017). Impact of sustainability labeling in the perception of sensory quality and purchase intention of chocolate consumers. J. Clean. Prod..

[87] Gaskell G., Hohl K., Gerber M.M. (2017). Do closed survey questions overestimate public perceptions of food risks?. J. Risk Res..

[88] Bawa A., Anilakumar K. (2013). Genetically modified foods: Safety, risks and public concerns—A review. J. Food Sci. Technol..

[89] Frewer L.J., van der Lans I.A., Fischer A.R., Reinders M.J., Menozzi D., Zhang X., van den Berg I., Zimmermann K.L. (2013). Public perceptions of agri-food applications of genetic modification—A systematic review and meta-analysis. Trends Food Sci. Technol..

[90] Wunderlich S., Gatto K.A. (2015). Consumer perception of genetically modified organisms and sources of information. Adv. Nutr..

[91] Frewer L.J., Bergmann K., Brennan M., Lion R., Meertens R., Rowe G., Siegrist M., Vereijken C. (2011). Consumer response to novel agri-food technologies: Implications for predicting consumer acceptance of emerging food technologies. Trends Food Sci. Technol..

[92] Fatimah U.Z.A.U., Boo H.C., Sambasivan M., Salleh R. (2011). Foodservice hygiene factors—The consumer perspective. Int. J. Hosp. Manag..

[93] Ko W.-H. (2010). Evaluating food safety perceptions and practices for agricultural food handler. Food Control.

[94] Kemp K., Insch A., Holdsworth D.K., Knight J.G. (2010). Food miles: Do UK consumers actually care?. Food Policy.

[95] Newsome R., Balestrini C.G., Baum M.D., Corby J., Fisher W., Goodburn K., Labuza T.P., Prince G., Thesmar H.S., Yiannas F. (2014). Applications and perceptions of date labeling of food. Compr. Rev. Food Sci. Food Saf..

[96] Castañé S., Antón A. (2017). Assessment of the nutritional quality and environmental impact of two food diets: A Mediterranean and a vegan diet. J. Clean. Prod..

[97] Skogen K., Helland H., Kaltenborn B. (2018). Concern about climate change, biodiversity loss, habitat degradation and landscape change: Embedded in different packages of environmental concern?. J. Nat. Conserv..

[98] Barrena R., Sánchez M. (2013). Neophobia, personal consumer values and novel food acceptance. Food Qual. Prefer..

[99] Fancello G., Paddeu D., Fadda P. (2017). Investigating last food mile deliveries: A case study approach to identify needs of food delivery demand. Res. Transp. Econ..

[100] Petrescu D., Petrescu-Mag R. (2015). Organic food perception: Fad, or healthy and environmentally friendly? A case on Romanian consumers. Sustainability.

[101] Ares G., Besio M., Giménez A., Deliza R. (2010). Relationship between involvement and functional milk desserts intention to purchase. Influence on attitude towards packaging characteristics. Appetite.

[102] Venter K., Van der Merwe D., De Beer H., Kempen E., Bosman M. (2011). Consumers’ perceptions of food packaging: An exploratory investigation in Potchefstroom, South Africa. Int. J. Consum. Stud..

[103] Ajzen I. (1991). The theory of planned behavior. Organ. Behav. Hum. Decis. Process..

[104] Grunert K.G., Aachmann K. (2016). Consumer reactions to the use of EU quality labels on food products: A review of the literature. Food Control.

[105] Power T.G., Johnson S.L., Beck A.D., Martinez A.D., Hughes S.O. (2019). The Food Parenting Inventory: Factor structure, reliability, and validity in a low-income, Latina sample. Appetite.

[106] Oude Ophuis P.A.M., Van Trijp H.C.M. (1995). Perceived quality: A market driven and consumer oriented approach. Food Qual. Prefer..

[107] Steptoe A., Pollard T.M., Wardle J. (1995). Development of a measure of the motives underlying the selection of food: The food choice questionnaire. Appetite.

[108] Peri C. (2006). The universe of food quality. Food Qual. Prefer..

[109] Hair J.F., Gabriel M., Patel V. (2014). AMOS covariance-based structural equation modeling (CB-SEM): Guidelines on its application as a marketing research tool. Braz. J. Mark..

[110] Tabachnick B.G., Fidell L.S. (2001). Using Multivariate Statistics.

[111] Comrey A.L., Lee H.B. (1992). A First Course in Factor Analysis.

[112] Jaeger S.R., Antúnez L., Ares G., Swaney-Stueve M., Jin D., Harker F.R. (2018). Quality perceptions regarding external appearance of apples: Insights from experts and consumers in four countries. Postharvest Biol. Technol..

[113] Bernués A., Olaizola A., Corcoran K. (2003). Extrinsic attributes of red meat as indicators of quality in Europe: An application for market segmentation. Food Qual. Prefer..

[114] Becker T. (2000). Consumer perception of fresh meat quality: A framework for analysis. Br. Food J..

[115] Glitsch K. (2000). Consumer perceptions of fresh meat quality: Cross-national comparison. Br. Food J..

[116] Brown C. (2003). Consumers’ preferences for locally produced food: A study in southeast Missouri. Am. J. Altern. Agric..

[117] Reichenberger J., Smyth J.M., Kuppens P., Blechert J. (2019). “I will fast … tomorrow”: Intentions to restrict eating and actual restriction in daily life and their person-level predictors. Appetite.

[118] Vermeir I., Verbeke W. (2006). Sustainable food consumption: Exploring the consumer “attitude–behavioral intention” gap. J. Agric. Environ. Ethics.

[119] Blackwell R.D., Miniard P.W., Engel J.F. (2001). Consumer Behavior.

[120] Kiel G.C., Layton R.A. (1981). Dimensions of consumer information seeking behavior. J. Mark. Res..

[121] Urbany J.E., Dickson P.R., Wilkie W.L. (1989). Buyer uncertainty and information search. J. Consum. Res..

[122] Annunziata A., Vecchio R. (2011). Factors affecting Italian consumer attitudes toward functional foods. AgBioForum.

[123] Liu R., Pieniak Z., Verbeke W. (2014). Food-related hazards in China: Consumers’ perceptions of risk and trust in information sources. Food Control.

[124] Carels R.A., Harper J., Konrad K. (2006). Qualitative perceptions and caloric estimations of healthy and unhealthy foods by behavioral weight loss participants. Appetite.

[125] Lazzarini G.A., Zimmermann J., Visschers V.H., Siegrist M. (2016). Does environmental friendliness equal healthiness? Swiss consumers’ perception of protein products. Appetite.

[126] Paquette M.-C. (2005). Perceptions of healthy eating: State of knowledge and research gaps. Can. J. Public Health/Revue Can. Sante Publique.

[127] Zhang Y., Tian Q., Hu H., Yu M. (2019). Water Footprint of Food Consumption by Chinese Residents. Int. J. Environ. Res. Public Health.

[128] Kilbourne W.E., Beckmann S.C. (1998). Review and critical assessment of research on marketing and the environment. J. Mark. Manag..

[129] Wardle J., Haase A.M., Steptoe A., Nillapun M., Jonwutiwes K., Bellisie F. (2004). Gender differences in food choice: The contribution of health beliefs and dieting. Ann. Behav. Med..

